# Improved Immunotherapy Efficacy by Vascular Modulation

**DOI:** 10.3390/cancers13205207

**Published:** 2021-10-17

**Authors:** Emma L. Newport, Ana Rita Pedrosa, Alexandra Njegic, Kairbaan M. Hodivala-Dilke, José M. Muñoz-Félix

**Affiliations:** 1 Centre for Tumour Microenvironment, Barts Cancer Institute, Queen Mary University of London, Charterhouse Square, London EC1M 6BQ, UK; e.l.newport@qmul.ac.uk (E.L.N.); rita.pedrosa@qmul.ac.uk (A.R.P.); a.njegic@qmul.ac.uk (A.N.); k.hodivala-dilke@qmul.ac.uk (K.M.H.-D.); 2 Department of Biochemistry and Molecular Biology, Institute of Biomedical Research of Salamanca (IBSAL), Universidad de Salamanca Spain, 37007 Salamanca, Spain

**Keywords:** immunotherapy, blood vessels, angiogenesis, vascular normalisation

## Abstract

**Simple Summary:**

Although immunotherapy has given the highest rate of improvement in cancer treatment in recent years, there is an urgent need to further improve its efficacy. Numerous strategies aim to transform non-responsive, immunosuppressive tumours into sensitive, immunopermissive tumours. The modulation of the tumour microenvironment, and especially the tumour vasculature offers opportunities for improved sensitivity to immunotherapy. Modulation of tumour blood vessels can enhance tumour oxygenation and T cell infiltration. Additionally, maturation of tumour blood vessels is thought to be involved in the efficient delivery of therapeutic agents. This review compiles the current strategies of vascular modulation to improve the efficacy of different immunotherapies: PD-1/PD-L1 and CTLA-4 antibodies, CAR T cells and cancer vaccines.

**Abstract:**

Several strategies have been developed to modulate the tumour vasculature for cancer therapy including anti-angiogenesis and vascular normalisation. Vasculature modulation results in changes to the tumour microenvironment including oxygenation and immune cell infiltration, therefore lending itself to combination with cancer therapy. The development of immunotherapies has led to significant improvements in cancer treatment. Particularly promising are immune checkpoint blockade and CAR T cell therapies, which use antibodies against negative regulators of T cell activation and T cells reprogrammed to better target tumour antigens, respectively. However, while immunotherapy is successful in some patients, including those with advanced or metastatic cancers, only a subset of patients respond. Therefore, better predictors of patient response and methods to overcome resistance warrant investigation. Poor, or periphery-limited, T cell infiltration in the tumour is associated with poor responses to immunotherapy. Given that (1) lymphocyte recruitment requires leucocyte–endothelial cell adhesion and (2) the vasculature controls tumour oxygenation and plays a pivotal role in T cell infiltration and activation, vessel targeting strategies including anti-angiogenesis and vascular normalisation in combination with immunotherapy are providing possible new strategies to enhance therapy. Here, we review the progress of vessel modulation in enhancing immunotherapy efficacy.

## 1. Introduction

### 1.1. The Advancement and Efficacy of Immunotherapies Give Hope

Immune modulation has provided a wide range of new potential therapies for many cancer types with some exciting advances in many malignancies [1,2,3]. Here, we explore the concept of tumour blood vessel modulation and the potential to improve response to immunotherapy. Solid tumours are heterogeneous and comprise cancer and stromal cells embedded within an extracellular matrix irrigated by an aberrant vasculature [4]. The dynamics between tumour cells and their environment is regulated by several cell types such as cancer-associated fibroblasts (CAFs) and tumour infiltrating leukocytes (TILs) [5].

It is well established that tumours are antigenic and induce a tumour specific immune response. For the host immune response to be effective, immune cells must simultaneously access the inside of the tumour and recognise it as ‘foreign’ to destroy it. The adaptive immune response comprises cytotoxic CD8+ cells, helper CD4+ T cells and antibody-producing plasma cells. Antigen binding to corresponding B or T cell receptors promotes the activation of the immune response as well as the generation of memory cells. Both CD8+ and CD4+ T cells recognise tumour antigens; CD8+ T cells play the main role in the antitumour response and CD4+ T cells differentiate into several types of helper CD4+ T cells [6]. Moreover, CD4+ T cells can differentiate into regulatory T cells (T_regs_), which can inhibit the host antitumour response. As such, T_regs_ are considered as an important target in cancer immunotherapy [5].

**Figure 1:**
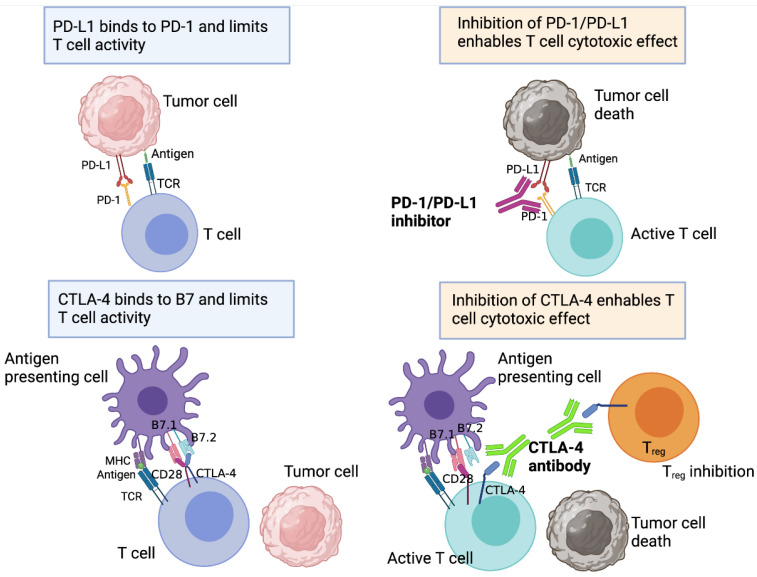
Anti-PD-1/PD-L1 and anti-CTLA4 immunotherapies basic principles. *Upper panels*: The interaction between tumour cell PD-L1 and T cell PD-1 inhibits the anti-tumour effect of T cells. Inhibition of PD-1/PD-L1 interaction activates T cells and promotes their anti-tumour effect. *Lower panels*: CTLA-4 competes with CD28 for B7.1/ B7.2 binding. CTLA-4 binding to B7.1/B7.2 maintains T cells in an exhausted state. Blocking this interaction using CTLA-4 antibodies activates T cells to induce their anti-tumour effect. Moreover, CTLA-4 inhibition also acts to enhance T cell priming and expan-sion in lymph nodes and at tumour sites by inhibiting T_reg_ activity. This potentiates its anti-tumour effect. Created with BioRender.com, accessed on 16 September 2021.

The emerging immunotherapy field is based on targeting mechanisms that are commonly used by cancer cells to evade the host immune response. For example, the use of mAbs directed against PD-1, PD-L1 and CTLA-4 have shown promising results in clinical trials [7] (Figure 1). PD-1, expressed by T cells, promotes the inhibition of T cell activity when it binds to ligands such as PD-L1, which is expressed on tumour and T_reg_ cells [8]. In fact, PD-1 binding to cancer cell PD-L1 can also activate survival signals further in-creasing the ability of cancer cells to evade T cells [9,10]. PD-L1 can bind CD28, thus regulating cytokine production and downregulating T cell activation [11]. Several PD-1 mAbs have been developed, including Nivolumab and Pembrolizumab, and they have shown efficacy in treating NSCLC [12]. Similarly, antibodies to PD-L1 such as Atezolizumab and Durvalumab have shown clinical benefit in NSCLC [13]. However, although PD1-PD-L1 interaction blockade has been generally successful in clinical trials for melanoma, NSCLC, renal cell cancer and ovarian cancer, the same treatments have not provided objective responses in other cancers including pancreat-ic cancer patients [14]. Although monotherapeutic immune checkpoint blockade for breast cancer has gener-ally not been as successful as expected, there are numerous clinical trials ongoing to determine the effect of this therapy in combination with radio and chemotherapies for breast cancer treatment (NCT04681287, NCT04418154). 

The most significant predictors of immunotherapy response include the PD-L1 status, tumour mutational burden (TMB), immune gene signatures, and the abundance of tumour infiltrating lymphocytes (TILs) [15,16,17]. In line with this, the majority of pancreatic ductal adenocarcinomas have poor immune cell infiltration [18] and the absence of a response in pancreatic cancer patients is thought to be due to the highly immunosuppressive TME which harbours a higher proportion of tumour-associated macrophages (TAMs), regulatory CD4+ T cells (T_regs_), myeloid-derived suppressor cells (MDSCs), in addition to the development of a dense desmoplastic stroma that is thought to be a physical barrier to immune infiltration [19]. In surgically resected pancreatic ductal adenocarcinoma (PDAC) patients, higher tumour infiltrating CD4+ and CD8+ cells can correlate with better survival [20]. Therefore, human whole cell granulocyte macrophage colony-stimulating factor (GM-CSF) secreting pancreatic cancer vaccines (GVAX) are being developed to increase PD-L1 expression and enhance tumour antigen-specific interferon-γ producing T cells in peripheral lymphocytes. In combination with anti-PD-1 vaccination, this approach has improved survival compared to either treatment alone [18].

Several immune checkpoint blockade strategies have shown efficacy. For example, CTLA-4, a protein expressed in T_reg_ cells and memory T cell membranes, was one of the first immune checkpoints studied [21]. CTLA-4 competes with CD28 for CD80, also known as B7.1, and CD86, also known as B7.2, ligands leading to the downregulation of T cell activation [22] (See Figure 1). Additionally, blockade of the PD-1/PD-L1 axis has offered consistent clinical improvement [23]. 

An alternative immunotherapy strategy consists of using chimeric antigen recep-tors (CARs) to redirect and reprogram patient T cells. CARs are genetically engineered synthetic receptors which provide T cells with the ability to target specific tumour surface antigens. Among others, CAR-T cells have been developed for lung cancer and malignant pleural mesothelioma treatment respectively [24,25]. Additionally, anti-CD-19 CAR-T cells have shown success in clinical trials for acute lymphoblastic leukaemia in both adults and children [26]. Importantly, CAR-T cells offer great promise in cancers with extremely poor prognosis. Multiple myeloma (MM), for example, was considered broadly incurable, but with current immunotherapy interventions, the MM specific B cell maturation antigen (BCMA) has proved to be a good target for CAR T cell therapy and clinical safety and ability to produce a short-term objective response are encouraging and extending the time to remission [27].

Apart from CAR T strategies, there are some therapies that can infiltrate T cells into the tumours and activate them such as the expansion of tumour-infiltrated lymphocytes (TIL) or transgenic TCR therapies. Expanded TIL therapy was set up in the 1980s when clinicians in the US developed a therapy by isolating and expanding tumour-infiltrated lymphocytes (TIL) hypothesising that ex vivo enrichment and expansion of them could provide a therapeutic benefit [28,29,30]. Recent studies have demonstrated that neoantigen specific T cells exhibit stronger responses, and this evidence has encouraged the identification of neoantigen targets in different cancers such as melanoma, lung cancer, gastric cancer, ovarian cancer and others [31]. Another strategy consists in the use of transgenic (Tg)TCR sequences. These TCR sequences can be captured from neoantigen-specific T cells and are being tested in preclinical models of acute myeloid leukaemia or solid tumours [32].

Other promising immunotherapies include cancer vaccines which prime the immune system to target one or more tumour-specific antigens. The aim of cancer vaccines is to induce tumour regression by stimulation of the patient’s adaptative immune response. They involve tumour antigens that activate dendritic cells [33]. For example, sipuleucel-T is an anti-cancer vaccine based on dendric cells loaded with prostatic acid phosphatase which is specific to prostate cancer cells. In 2010, it was the first cancer vaccine to be FDA approved [34].

### 1.2. Immunotherapy: Achievements and Challenges Focused on PD-1/PD-L1 and CTLA-4 Pathways

Immune checkpoints such as PD-1/PD-L1 and CTLA-4 pathways have introduced a new era in cancer treatment, with a number of drugs currently in Phase I-III trials.

Nivolumab is a fully human IgG4 programmed death 1 (PD-1) immune-checkpoint inhibitory mAb, that drives improvements in overall survival, response rate and progression-free survival in lung cancer. The beneficial effects of Nivolumab were shown to be significantly better than docetaxel, independent of PD-L1 levels [35]. Pembrolizumab, a humanised anti-PD-1 mAb of the IgG4 kappa isotype, blocks the interaction between PD-1 and the ligands PD-L1 and PD-L2 [36]. Pembrolizumab has shown an acceptable side-effect profile and strong anti-tumour activity in patients with advanced NSCLC. Moreover, the efficacy of Pembrolizumab correlated with PD-L1 levels in at least 50% of tumour cells [37]. On the other hand, Atezolizumab is a humanised anti PD-L1 mAb that inhibits its interactions with PD-1 and B7-1. Atezolizumab has demonstrated an improvement in overall survival when compared to docetaxel in previously treated NSCLC patients. This occurs without any correlation with PD-L1 expression, as shown in the first randomised Phase III study [38]. Apart from the mAbs targeting the PD-1/PD-L1 pathway, several anti-CTLA-4 mAb, such as Ipilimumab and Tremelimumab, are showing improvements in combinations with second-line chemotherapies [39]. It is necessary to mention the poor response observed with anti-CTLA-4 monotherapy, as demonstrated using Ipilimumab in metastatic melanoma. However, there is a strong additive response when using in combination with anti-PD-1 therapy [40].

Patients who do not respond to immunotherapies normally lack cytotoxic immune cell infiltration in their tissues and exhibit variable PD-L1 expression on their tumour cells which can impair the efficacy of immunotherapies. Elevated levels of immunosuppressive cells (T_regs_, myeloid-derived suppressor cells (MDSCs) and TAMs) are present in both pancreatic cancer patients [41] and mouse models of pancreatic cancer [42]. T_regs_ produce an immunosuppressive TME and are actively recruited to pancreatic cancers [43]. T_reg_ levels are increased in both the tumour and in the circulation [44,45], and this is associated with progression of disease and worse prognosis [45,46]. Impaired delivery of mAbs in several tumours could limit the efficacy of immunotherapy treatment. 

Increasing immune infiltration to the tumour is another feature that is considered to be essential for immunotherapy efficacy. The CXCR4 antagonist BL-8040 has shown promise pre-clinically for its ability to mobilise immune cells in PDAC, specifically CD4+, activated CD8+ T cells and dendritic cells and is now in clinical trials in combination with pembrolizumab and chemotherapy for pancreatic cancer: the COMBAT trial or combination with the anti-PD-L1 mAb Atezolizumab (NCI-2017-01495). 

Although the efficacy of immune checkpoint inhibitors (ICI) has been explored and demonstrated in monotherapy, current clinical trials consider the use of ICI in combination with chemotherapy and immunotherapy [35,38,47,48,49,50]. Numerous studies have demonstrated the induction of an immunopermissive tumour microenvironment by the chemotherapeutic agents. Chemotherapy has the ability to increase the number of CD8+ T cells, enhance the levels of PD-L1, promote the maturation of antigen presenting cells and the downregulation of immunosuppressive cells such as T_regs_ or MDSCs [51,52]. This modification of the tumour microenvironment is helpful to enhance the efficacy of immunotherapy. Based on this evidence, numerous clinical trials are assessing the efficacy and safety of chemotherapies with ICI [53,54,55,56]. 

Recent studies have also demonstrated the synergy of radiotherapy (RT) and immune checkpoint blockade in preclinical models [57]. Similar to chemotherapy, radiotherapy also promotes an immunopermissive tumour microenvironment [58]. The impact of radiation on the vasculature has been extensively reviewed previously [59,60]. Overall, low radiation dosages seem to have a pro-angiogenic effect (<2 Gy), while higher dosages seem to be anti-angiogenic. Importantly, radiation induces endothelial cell activation, causing the quiescent phenotype to switch to a proinflammatory state, characterised by activation of the genotoxic stress-induced nuclear factor (NF)- kB pathway and expression of chemokines, cytokines and adhesion molecules that facilitate the recruitment and attachment of both leucocytes and tumour cells on the vascular wall [61]. However, when exposure is repeated and/or prolonged its exhausts the protective physiological effects of the endothelium, leading to endothelial cell dysfunction with consequent deterioration of the vascular tone, blood haemostasis problems, inflammation and oedema at the sites of the affected endothelium. As mentioned before, depending on the radiation dose, quality and intrinsic sensitivity of the vascular bed, ionising radiation can also cause endothelial cell death, therefore compromising the endothelial barrier and delivery of oxygen, nutrients and possible therapeutic drugs administered systemically [59]. A few reports suggest that after ablative radiation dosages, the vasculature can be restored by vasculogenesis aided by immune cells: Kuonen and colleagues have shown that hypoxia, induced by irradiation anti-angiogenic effects, drives expression of CXCL12 promoting mobilisation from the bone marrow and recruitment to primary tumour sites of CXCR4+CD11b+ bone marrow-derived cells that assist vasculogenesis [62]; In experimental pre-clinical models, restoration of the vasculature after RT is mediated by irradiation induced influx of bone marrow derived monocytes and macrophages, which eventually contributes to tumour regrowth [63,64,65]. Conversely, the direct effects of radiation on the vasculature have also been shown to polarise macrophages towards an M2 phenotype- this effect was shown to be dependent on CXCR4 (SDF-1 receptor) upregulated in irradiated ECs [66]. A very recent study has shown the beneficial effects on restricting tumour progression using a combination of vascular targeting, hypofractionated radiotherapy (administering higher dosages in less frequent fractions), and immune checkpoint inhibitor [67]. The authors have used a TEM1 (Tumour endothelial marker1 is a protein expressed in the tumour-associated endothelium) vaccine together with RT and anti-PD1 antibody in subcutaneous colorectal and lung tumours and shown an effective anti-tumour response. Mechanistically, RT increased MHCI expression in ECs, thus improving immune recognition with consequent vascular damage by anti-TEM1 T cells. The TEM1 vaccine combined with RT boosted tumour-associated antigen (TAA) cross-priming driving increased PD-1/PD-L1 signalling. Additional blockade of PD-1/PD-L1 axis further boosted the dual-therapy anti-tumour effect and TAA immune responses [67]. Of note, combination of immune checkpoint inhibitors with different radiotherapies: external beam radiation therapy, intensity-modulated radiation therapy or stereotactic body radiation therapy [68] are currently being tested in clinical trials. 

### 1.3. Tumour Infiltrated Leukocytes (TIL) as A Prognostic Marker

Tumours poorly infiltrated by T cells, or tumours where this infiltration is not observed in the core of the tumour mass, generally do not respond to immunotherapies [69]. In lung cancer, several studies have demonstrated a correlation between CD8+ TILs and prognosis. In some studies, high CD4+/CD8+ infiltration correlates with longer overall survival [70,71]. Moreover, T_regs_, Cox2 or mature dendritic cells have been correlated with prognosis of recurrence in NSCLC [72], although a more recent study failed to find a link between the CD8+ infiltrate and a better prognostic value, finding only an association with better outcome [73]. To overcome the limitation of TIL infiltration, modulating the tumour vasculature is an interesting approach.

## 2. Tumour-Associated Vasculature

One of the hallmarks of tumour development and dissemination is angiogenesis [74]. Due to the metabolic demands of the tumour cells together with the diffusion limitations of both oxygen and nutrients from pre-existing vessels, for a tumour mass to grow beyond 1–2 mm^3^ it requires the triggering of an angiogenic switch. This switch is induced when a tumour produces sufficient pro-angiogenic growth factors and/or suppresses the expression of anti-angiogenic molecules [75].

Tumour blood vessels, particularly those in the inner regions of the tumour, are structurally and functionally abnormal. Structurally, these vessels exhibit a highly disorganised architecture, irregular diameters along their length, tortuous branching, oversized pores and an absence of mural cell (pericytes and smooth muscle cells) coverage. These structural abnormalities lead to functional changes, characterised by irregular and slow blood flow with increased vessel permeability and leakiness. This form of vasculature leads to an inadequate supply of nutrients and oxygen and inefficient delivery of cancer therapeutics. We will describe all these mentioned features and the cellular and molecular mechanisms in the following sections.

### 2.1. Mechanisms of Tumour Neovascularisation

Tumours become vascularised through a combination of new vessel development and exploitation of the surrounding vasculature from healthy tissue. Angiogenesis is perhaps the most well understood mechanism of tumour vascularisation and is a target for therapy.

#### 2.1.1. Angiogenesis

The formation of new blood vessels from the pre-existing vasculature is the main method of neovascularisation in tumours with their hypoxic and necrotic regions acting as inducers of angiogenesis.

When a capillary receives an angiogenic stimulus, endothelial cell basal membrane and extracellular matrix are degraded, releasing endothelial cells from their basement membrane anchors (including integrins). This process is mediated by metalloproteases, leading to disruption of tight junctions, vasodilation and pericyte detachment. Existing soluble growth factors coupled with the synthesis of a new matrix by stromal cells enable the migration and proliferation of endothelial cells [75]. Migration is performed by specialised endothelial tip cells and the direction of migration is mainly determined by vascular endothelial growth factor A (VEGF-A) [76]. Endothelial sprouts form solid migrating columns, which can be remodelled into a lumen when endothelial cells adhere to each other and inter-endothelial tight junctions are re-established. Newly formed capillaries are stabilised and matured by recruitment of perivascular cells (pericytes and smooth muscle cells). Ultimately, if proper angiogenesis occurs, blood flow is established in the newly vascularised area [75].

#### 2.1.2. Vessel Co-Option

There is evidence that both primary tumours and metastases can progress without angiogenesis [77]. Vessel co-option is a mechanism in which tumours receive a blood supply by hijacking the pre-existing vasculature and this can be the main mode of tumour metastasis support in breast cancer to the lung and brain and colon cancer to the liver [78,79,80,81,82]. Additionally, this is seen in primary tumours of highly vascularised organs, such as lung and liver [81]. Some other evidence suggests that tumour cells can compress and destabilise the pre-existing vasculature, activating a host defence mechanism which initiates an apoptotic response leading to regression of co-opted vessels and perfusion impairment [83]. This ultimately leads to hypoxia and necrosis triggering further neovascularisation, so a combination of neovascularisation and co-option can exist together [84]. 

Vessel co-option has been suggested as a mechanism of resistance to anti-angiogenic therapy [83]. Preclinical models of pulmonary metastases display vessel co-option after anti-VEGF therapy [84]. On the other hand, it was shown that in human patients with colorectal cancer liver metastasis, vessel co-option appears in those with poor response to the anti-angiogenic drug bevacizumab [85].

### 2.2. Cellular and Molecular Mechanisms Controlling Angiogenesis

#### 2.2.1. HIF1α

As tumours grow, nutrient and oxygen supplies become insufficient causing tumour necrosis, especially in the central part of the growing mass. Together with increased metabolic waste accumulation, acidosis and hypoxia soon develop. Tumour hypoxia is the major driver of neovascularisation. Under hypoxic conditions, hypoxia-inducible factor-1 (HIF1α) accumulates and binds to hypoxia response elements (HREs), a DNA sequence motif present in HIF target genes. Among these are numerous genes involved in cellular responses to hypoxia, such as metabolic reprogramming and angiogenesis which are typically markers of poor prognosis [86,87]. Thus, hypoxia stimulates the secretion of both pro-angiogenic factors, i.e., VEGF-A, angiopoietins, fibroblast growth factor (FGF), platelet-derived growth factor (PDGF) and TNF-α, and anti-angiogenic factors, i.e., angiostatin, endostatin, interferons, tumour necrosis factor-β and thrombospondins.

#### 2.2.2. VEGF

The VEGF signalling cascade is considered the main pathway for regulation of angiogenesis. VEGF-A, a pro-survival factor, is considered the main mediator in hypoxia-induced tumour growth [88], it inhibits endothelial cell apoptosis [89], and regulates vascular permeability and vessel dilation by loosening inter-endothelial tight junctions [90]. More specifically, VEGF-A exerts its pro-angiogenic biological function by interacting with VEGFR-2, a transmembrane receptor that belongs to the superfamily of receptor tyrosine kinases (RTK) located on endothelial cell membranes. Ligand–receptor interaction causes their dimerisation, triggering trans/auto-phosphorylation of tyrosine residues in the cytoplasmic kinase domain which stimulates a number of signal transduction pathways that drive mitogenesis, migration and survival of endothelial cells [91]. However, despite its importance in angiogenesis, there is redundancy in VEGF-A-driven tumour angiogenesis which is one possible reason why tumours can become resistant to VEGF-A targeted anti-angiogenic therapy [92].

#### 2.2.3. Angiopoietins

Another signalling system involved in the balance between quiescent and activated endothelium, particularly in the regulation of interactions between endothelium and surrounding support cells, is the tyrosine kinase receptor Tie-2 (Tek) and its ligands—angiopoietins (Ang). Angiopoietin 1 is expressed by mesenchymal cells, including pericytes and smooth muscle cells, while Tie-2 is expressed on the surface of endothelial cells [93]. Consequently, Ang-1/Tie-2 signalling promotes the association between endothelial cells and perivascular support cells, contributing to stabilisation of the maturing vascular system and decreased vascular permeability. Opposingly, Ang-2 is a competitive inhibitor, or antagonist, of Ang-1. Ang-2 binds to Tie-2 on endothelial cells leading to weakened tight junctions and pericyte dissociation, destabilising blood vessels and acting as a cooperative driver of angiogenesis [94,95]. However, in the presence of VEGF-A, Ang-2 is able to bind integrins leading to sprouting angiogenesis [96]. 

#### 2.2.4. PDGF

Platelet-derived growth factor (PDGF) promotes the survival, proliferation and migration of cells of mesenchymal origin [97]; the PDGF signalling pathway is also involved in recruitment of pericytes to newly formed vessels [98]. During angiogenesis, endothelial cells, particularly tip cells, express Pdgf-b, which exerts a mitogenic effect on pericytes/vSMCs expressing Pdgfr-β, driving proliferation, directed migration and incorporation into the vessel wall [93]. When vascular smooth muscle cells (vSMCs) and pericytes are attracted, they tightly encircle and associate with the endothelium producing survival and anti-proliferative factors that stabilise nascent vessels [99]. Dysfunction of PDGF signalling is common in cancer and is therefore considered a potential drug target [97].

#### 2.2.5. Integrins

Integrins are a family of heterodimeric transmembrane glycoproteins that mediate cell–cell and cell–ECM interactions. The integrin family consists of eight β- and 18 α- subunits that assemble as heterodimers to form 24 distinct integrins [100]. Each integrin heterodimer binds a specific set of endogenous ligands, which include ECM components (such as collagen, fibronectin and vitronectin), soluble ligands and ligands on the cell surface such as VCAM-1 (Vascular cell adhesion molecule-1) or ICAM-1 (Inter cellular adhesion molecule-1). Each integrin subunit consists of an extracellular domain, a transmembrane region and a short cytoplasmic region that links them to the cytoskeleton. Therefore, integrins are able to transduce signals across the plasma membrane in both directions. Upon ligand binding, integrins cluster and activate intracellular signal transduction that promotes endothelial cell migration, proliferation and survival [101].

Endothelial cells express up to ten different integrins: α1β1, α2β1, α3β1, α4β1, α5β1, α6β1, α6β4, αvβ3, αvβ5 and αvβ8 [102]. Of these, during angiogenesis, integrins α5β1, α2β1 and α1β are upregulated, promoting cell migration, proliferation and matrix reorganisation [103]. Additionally, it has also been shown that αv integrins play important roles in angiogenesis, with αvβ3 being selectively expressed on proliferating endothelial cells. Results from studies of integrin antagonists indicate that αv integrins (αvβ5 and αvβ3) promote angiogenesis [104,105,106,107,108,109,110,111]. However, genetic ablation studies indicate that αvβ5 and αvβ3 integrins are not required for angiogenesis, because loss of αvβ3 enhances VEGF-receptor 2 expression [112,113]. Indeed, low doses of αvb3/αvb5-specific RGD-mimetic, Cilengitide, can actually enhance tumour angiogenesis [114] and this feature can be used to enhance the efficacy of gemcitabine treatment in non-small cell lung cancer and pancreatic ductal adenocarcinoma models in mice [115,116,117]. 

### 2.3. Vascular Regulation of the Immune Infiltrate: Endothelial Anergy

The roles of endothelial cells and angiogenic factors are closely linked with immunity and inflammation. Angiogenic factors in the TME promote immunosuppressive features [118,119,120]. Increasing types of immune cells are being identified as promoting both immunosuppression and angiogenesis, including T_regs_ [121], myeloid-derived suppressor cells (MDSCs) [122], natural killer (NK) cells [123] and neutrophils [118,124]. These cells also increase the production of VEGF-A [118] which, in addition to its role driving tumour angiogenesis, also potently suppress both the innate and adaptive anti-tumour immunity. It inactivates NF-kB signalling preventing dendritic cells from maturing and presenting antigens to T cells in mouse models [125,126,127]. The T cell response is further depleted as VEGF inhibits their development and upregulates immune checkpoints preventing T cell activation [128,129].

Under normal, non-inflamed, conditions blood vessels are quiescent. Due to minimal surface expression of adhesion proteins, leukocytes are prevented from adhering. However, in response to proinflammatory cytokines, such as TNF, IFNγ and IL-1, endothelial cells are activated and adhesion proteins are trafficked to the surface. This allows leukocytes to extravasate as ICAM1 surface expression is sufficient for their migration out of blood vessels [120,130,131].

In response to angiogenic factors, tumour blood vessel endothelial cells change phenotypically, and one feature of this is the downregulation of adhesion molecules expression such as ICAM-1, making the endothelial cells unresponsive to inflammatory cytokines and increasing expression of immune inhibitory molecules. Together these changes create a barrier for immune cells in a process known as endothelial cell anergy [120,132].

The promotion of the immunosuppressive tumour microenvironment by ECs occurs in several directions. First, the upregulation of molecules such as common lymphatic endothelial and vasculature endothelial receptor 1 (CLEVER1) has been shown to promote the accumulation of T_regs_ [133]. Second, tumour ECs upregulate receptors which limit T cell activity, such as PD-L1, PDL-2 or TIM3 [134,135,136]. Other inhibitory molecules such as IL-6, PGE2, IL-10 and TGFβ have been shown to be secreted by ECs within the tumour [137]. Apart from these cytokines, the induction of apoptosis by ECs is promoted by the secretion of molecules such as TRAIL and FasL [138].

In the tumour setting, endothelial cells are continuously exposed to high levels of angiogenic growth factors, become unresponsive to proinflammatory cytokines and thus express fewer adhesion proteins. Therefore, continued angiogenesis is thought to prevent effective leukocyte infiltration [120,139,140,141]. Endothelial anergy has been described as the vascular counterpart of immune checkpoint as both are important regulators of immune homeostasis. Physiological angiogenesis often takes place in concert with immunosuppression, in response to the same stimuli [120,142]. It has therefore been proposed that exploiting the vascular normalisation effect of some anti-angiogenics may increase leukocyte infiltration and thereby immune checkpoint immunotherapy efficacy [120,143,144,145].

## 3. Strategies for Tumour Vasculature Modulation and Improved Immunotherapy

### 3.1. Improving Immune Checkpoint Blockade: Anti-Angiogenic Therapy, Vascular Normalisation and Induction of High Endothelial Venules (HEVs)

#### 3.1.1. Anti-Angiogenesis and Vascular Normalisation

**Figure 2:**
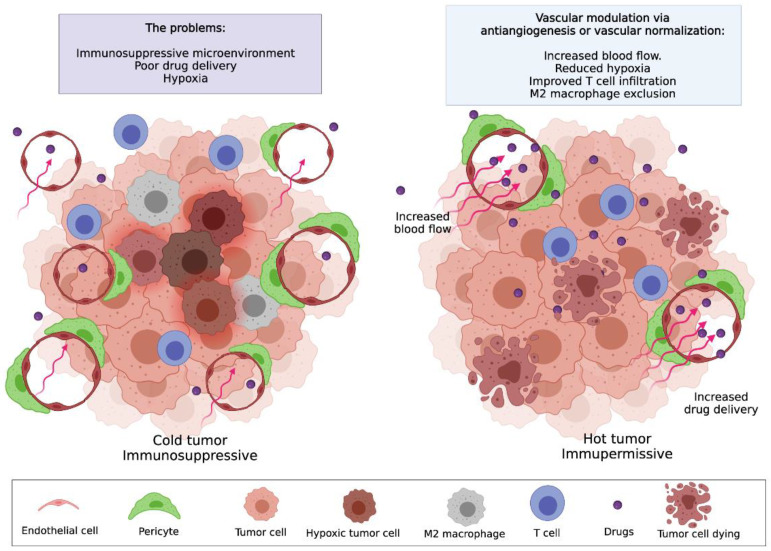
Modulating tumour vasculature to enhance immunotherapy efficacy. Immune checkpoint blockade is not effective in many cancer types due to the hypoxic immunosuppressive tumour microenvironment including endothelial cells of tumour blood vessels. Anti-angiogenic therapy reduces blood vessel density and some of the immunosuppressive effects of tumour angiogenesis. Anti-angiogenesis and vascular normalisation, using low dose anti-angiogenic drugs, reduces the immunosuppressive tumour microenvironment by increasing blood flow, and drug delivery and reducing subsequent hypoxia, making favourable conditions for the infiltration of CD8+ cells and allowing the effectiveness of immune checkpoint blockade. Created with BioRender.com, accessed on 16 September 2021.

Over the last 20 years, a wide variety of anti-angiogenic compounds have been developed for solid tumours. These drugs prevent the formation of new blood vessels by angiogenesis, and although they have not been as successful as hoped when used as monotherapies [146,147], they have met considerable success when used in combination with chemotherapy or immunotherapy [10,148,149]. Through their role in preventing angiogenesis, many of these drugs also promote the maturation of blood vessels especially when used at low doses. This concept, that lower doses of anti-angiogenic drugs can ‘normalise’ the tumour vasculature is referred to as vascular normalisation [150]. Low-dose anti-angiogenic therapy increases pericyte coverage which improves vessel stabilisation, reducing vascular permeability and leakiness [151,152]. Vascular normalisation reduces hypoxia and increases the delivery and efficacy of cytotoxic agents, overcoming the important limitations observed in anti-angiogenic therapy. This normalised vasculature also reduces immunosuppression through depletion of T_regs_ and regulatory B cells, enhancement of M1 TAMs and activation of T cells (especially CD8+) [153] (see Figure 2). Administration of low dose DC101, a VEGFR2 inhibitor, in combination with a mitomycin C vaccine leads to a higher infiltration of CD4+ and CD8+ cells and lower levels of MDSCs and T_regs_, resulting in decreased hypoxia, breast cancer cell proliferation and increased survival [154]. Many of these anti-angiogenics and vascular normalisation drugs are now being trialled in combination with immunotherapy, in some cases, the anti-angiogenics and immunotherapy are documented to work synergistically together [10].

DC101 enhances the anti-tumour response of a cancer vaccine targeting neu (the murine equivalent of HER2) gene expression in an immunocompetent mouse model of tumours driven by over expression of neu [10,155]. In this model, DC101 alone decreased angiogenesis and increased tumour cell apoptosis. Despite the reduction in angiogenesis, the numbers of tumour infiltrating lymphocytes were increased two weeks after DC101 treatment of tumour-bearing mice without vaccination. In fact, the reduced tumour growth induced by DC101 was found to be in part through enhanced anti-tumour immunity with increased tumour specific CD8+ T cells [155]. The reason for the enhanced immunity in these DC101 treated animals was hypothesised to be due to a vascular normalisation effect, as DC101 has been shown to promote the pruning of immature vessels and the maturation of the remaining ones [156].

Immune checkpoint inhibitors have also shown promise in combination with anti-angiogenics. In melanoma patients, increased levels of VEGF in the serum before treatment with the anti-CTLA4 immune checkpoint blockade antibody ipilimumab is predictive of worsened overall survival. In addition, in melanoma brain metastases, treatment with anti-angiogenics reduces the immunosuppressive microenvironment. Therefore, the combination of anti CTLA-4 with axitinib, an inhibitor of VEGFR1, 2 and 3 was tested in subcutaneous and intracranial mouse models of melanoma. Combination of anti-CTLA-4 with axitinib treatment increases the numbers of effector T cells and antigen presentation by intratumoural dendritic cells while reducing the immunosuppression of MDSCs and improving animal survival compared to axitinib alone [10,157] suggesting a very attractive strategy for improving immunotherapy. Additionally, anti-PDL1, Atezolizumab, when combined with bevacizumab plus chemotherapy significantly improved progression-free survival and overall survival among patients with metastatic NSCLC [158].

The multi-kinase inhibitor Sorafenib has anti-angiogenic properties and has been used for advanced hepatocellular carcinoma (HCC) treatment since 2007. Sorafenib inhibits the molecular components of the Raf–MEK–ERK signalling pathway, inhibiting tumour growth and VEGFR-1, VEGFR-2, VEGFR-3 and PDGFR-β, thereby inhibiting angiogenesis [159,160]. This suggests that HCC responds to anti-angiogenic treatment. The recent IMbrave150 phase III clinical trial, which combined an anti-PD-L1 checkpoint blockade inhibitor, Atezolizumab, and the anti-angiogenic drug, Bevacizumab, demonstrated that combination therapy improved overall survival compared to Sorafenib monotherapy. This combination therefore gained FDA approval in 2020 for the treatment of patients with unresectable or metastatic HCC who have not received prior therapy. This combination of anti-angiogenesis and immunotherapy was a significant breakthrough as there had been little advance in the treatment of HCC [161].

#### 3.1.2. Induction of HEVs

High endothelial venules (HEVs) are blood vessels especially adapted for lymphoid cell trafficking and are normally found in secondary lymphoid organs such as lymph nodes and Peyer’s patches. Additionally, they can develop in non-lymphoid organs during chronic inflammation driven by autoimmunity, infection or allografts. More recently, HEVs have been observed in solid and vascularised tumours, and their presence correlates with a reduced tumour size and good prognosis [162] making them a target for intervention in the enhancement of immunotherapy. HEVs regulate the adhesion, migration and activation of circulating T and B cells. Tumoural HEVs have been associated with improvement in patient outcomes, suggesting further that therapeutic HEV induction in solid tumours could be a potential tool to enhance T cell infiltration and sensitise tumours to immunotherapy. It has been shown that the combination of immunotherapy (anti-PD-L1) and anti-angiogenic therapy (anti-VEGFR2) increases HEV formation in breast and pancreatic experimental tumours, enhancing T cell infiltration [162]. Recently, it has been demonstrated that the administration of LIGHT (Tumour necrosis factor superfamily member 14) in experimental glioblastoma models normalises tumour vasculature and induces HEV formation. The normalisation of the angiogenic blood vessels, together with the induction of HEV formation, leads to an increase in T cell infiltration, which is further amplified in combination with PD-1 therapy [163].

### 3.2. Improving the Efficacy of CAR-T Cell-Based Immunotherapy

CAR T cells activity is affected by the vascularity of the tumour microenvironment and also has the capability to modulate it. As with other immunotherapies, the efficacy of CAR T cell immunotherapy is closely linked with the microenvironment. For example, high levels of hypoxia impair the expansion and differentiation of CAR-T cells into effector memory cells in vitro [164,165]. In order for therapy to be effective, CAR-T cells must infiltrate the solid tumour and have cytotoxic activity. It is thought that hypoxia in solid tumours may allow immune escape and prevent CAR-T cell-mediated cytotoxicity. This is proposed as a possible reason for the often disappointing results of CAR-T cell therapy in the clinic [165,166]. This field has so far been hindered by a lack of in vitro models that accurately model the complexity of the tumour microenvironment; novel tools are therefore required. For example, a microfluidic device developed by Ando et al. recapitulates the oxygen gradient across a solid tumour section. Using this model, they demonstrated differential killing by CAR-T cells at different oxygen tensions which may be useful in future in vitro preparation of CAR-T cells [166].

#### 3.2.1. *Careful Investigation of the Impact of Hypoxia on CAR-T Cells Is Important*

The downstream effects of CD28 and 4-1BB show different effects on cell metabolism. CAR T cells with 4-1BB show enhanced respiratory capacity, while CAR T cells with CD28 domains show increased glycolysis [165,167,168,169]. It is therefore important to consider the state of oxygenation of the solid tumour, and consequently, modulation of the vasculature could possibly be a useful tool to ensure the best level of oxygenation for the CAR-T cell-based therapy. An alternative approach looks at using the level of hypoxia to direct CAR T cells activity. Under normal circumstances, hypoxia may pose challenges to the activity of CAR T cells, but many solid tumour environments are highly hypoxic in contrast to surrounding organs. Therefore, manipulation of CAR T cells to make them active under hypoxic conditions is a valuable target which may allow their activity to be directed to the tumour rather than oxygenated, healthy microenvironments. This can be achieved by either targeting CAR T cells specifically to antigens present in hypoxic microenvironments such as carbonic anhydrase IX [170] or by adding regions to the CAR T cells such as a HIF domain that will promote its hydroxylation and degradation in oxygenated environments [165,171]. These strategies in CAR T cells are interesting in that they may work best in hypoxic conditions, possibly lending themselves to combination with anti-angiogenics. However, in poorly vascularised tumours, there will be adverse effects of systemic CAR T cell delivery; Cui et al. propose that these may be mitigated by direct injection of the CAR T cells into the tumour site [170].

Immunotherapy can also be used directly against the tumour vasculature. Human prostate-specific membrane antigen (hPSMA) is present on the tumour endothelial cells of many solid tumours but absent from healthy vasculature [172,173]. CAR-T cells engineered against PSMA, known as P28BBζ CAR-T cells, recognise and kill PSMA^+^ tumour endothelial cells in vitro including primary human endothelial cells isolated from gynaecologic cancers. In mouse models of ovarian cancer with vessels expressing hPSMA, the P28BBζ CAR T cells successfully ablate PSMA+ vessels, resulting in a depletion of tumour cells and reduced tumour burden through their anti-angiogenic effects [4,174]. In addition to CAR-T cells targeted against PSMA, they have also been targeted against the tumour blood vessel markers VEGFR-2 [175], VEGFR1 [176], TEM8 [177] and fibronectin splice variant EIIIB [165,178]. CAR-T cells against each of these antigens are capable of destroying tumour vasculature and have shown promising results pre-clinically. However, a clinical trial of CAR-T cells against VEGFR-2 in metastatic cancer patients that had not responded to, or relapsed after, standard treatment, had to be discontinued due to a lack objective responses (NCT01218867). 

It is possible that CAR-T cells developed to target the blood vessels could be used in combination with CAR-T cells targeted directly against the tumour. For example, CARs against VEGFR-2 have been used in combination with transgenic TCR with specificity against the murine melanoma antigens gp100 and tyrosinase-related protein-1 (TRP-1). In mice bearing subcutaneously injected B16 melanomas, anti-VEGFR-2 CAR-T cells did not enhance the antitumour affect alone but in combination with CARs targeted against melanoma; they worked synergistically to increase intra-tumoural T cells, eradicating B16 tumours and extending progression free survival [4,175,179]. The authors previously demonstrated that, 4 days after adoptive cell transfer (ACT) there was a reduced number of CD31+ endothelial cells compared with those treated with empty vector-transduced cells [175] but could not quantify the microvessel density due to high levels of tumour necrosis. They offer two possible hypotheses for the synergistic enhancement of CAR-T cell therapy in this study. They suggest that pruning of blood vessels by the anti-VEGFR-2 CAR-T cells may promote vascular normalisation in the remaining blood vessels [150,180] more than compensating for the lost vessels in terms of drug and possibly immune cell delivery [150]. Alternatively, the anti-VEGFR-2 CAR-T cells may act to reduce the populations of immunosuppressive cells including T_regs_ and MDSCs which express VEGFR-2 [122,179,181,182,183,184,185,186].

#### 3.2.2. Vascular Modulation for Improved Cancer Vaccines Efficacy

Similar to CAR-T cells, cancer vaccines have been produced against tumour blood vessels to promote an anti-angiogenic effect [187,188,189]. In addition, cancer vaccines have been used in combination with anti-angiogenic treatments. For example, the tyrosine kinase inhibitor sunitinib has been used in combination with a dendritic cell-based vaccine expressing IL-12 and pulsed with ovalbumin (OVA) protein in a B16-OVA mouse model of melanoma. Sunitinib improved the anti-tumour T cell infiltration and reduced the protumourigenic immune cells such as T_regs_ and MDSCs and the same results could be replicated with an alternative anti-angiogenic tyrosine kinase inhibitor, axitinib. The timing of the anti-angiogenic agent in these studies was found to be important with best results when the drug was administered at the same time as the vaccine and poorer results when it was administered after [190,191,192].

Huang et al. demonstrated that a mitomycin C-pre-treated MCaP0008 cancer vaccine stimulated splenic CD8+ T cells to produce IFN-γ, but it did not inhibit tumour growth in mice bearing orthotopic MCaP0008 breast cancer, suggesting immune tolerance. However, in combination with the anti-angiogenic DC101 at low, vascular normalising doses, tumour growth was significantly inhibited. The exact dose of DC101 was shown to be important in this combination [154]. When used as a monotherapy, in pre-clinical models, DC101 is only effective at the full, anti-angiogenic dose of 40 mg/kg but there does not delay tumour growth at the vascular normalising dose of 10 mg/kg [143,150,193]. In contrast, in combination with the vaccine, at 10 mg/kg, tumour growth was inhibited but at 40 mg/kg, there was no improvement with the combination compared to DC101 alone. with this low dose of DC101, there was improved tissue perfusion, more immunostimulatory compared to immunopermissive cells and, in the MMTV model, better survival demonstrating that the enhanced vaccine effects were due to vascular normalisation [154,192].

Despite some promising results preclinically, combinations of cancer vaccines with anti-angiogenic therapies have met limited success in clinical trials. For example, sunitinib was combined with a personalised DC based vaccine (AGS-003) for the treatment of metastatic renal cell carcinoma. In a phase II clinical trial, administering AGS-003 at the start of the second sunitinib cycle resulted in an improved immunological response and prolonged survival [194]. However, in a phase III trial of the same combination, AGS-003 failed to improve OS compared to sunitinib alone [195]. It is thought that more work may be needed to optimise the correct dose and timing of the anti-angiogenic therapy when used in combination with cancer vaccines [192]. However, several trials are still ongoing or awaiting results including combination of Bevacizumab with personalised cancer vaccine for recurrent glioblastoma multiforme [196] and intratumoural vaccination pre-nephrectomy combined with post-nephrectomy sunitinib for the treatment of newly diagnosed metastatic renal cell carcinoma [197].

## 4. Conclusions

The rise of immunotherapies has shown very promising results in clinical practice giving huge benefits in cancer treatment especially in combination with chemo/radiotherapy. Recent work now shows that that modulation of tumour blood vessels in combination with immunotherapy provides further advancement in the efficacy of immunotherapies. Further studies will be required to explore the complex dosing and scheduling priorities required and how best to mitigate toxicity issues. Future confirmation of whether such multimodal combination therapies, including vessel modulation, will be effective across multiple cancer types, especially those of unmet clinical need, raises excitement and hope for the further improvement in cancer treatment.
